# Supplementary material for: NR-SAFE: a randomized, double-blind safety trial of high dose nicotinamide riboside in Parkinson’s disease
Source: Nat Commun. 2023 Nov 28;14:7793. doi: 10.1038/s41467-023-43514-6 (PMC10684646; doi:10.1038/s41467-023-43514-6)
Supplement: Supplementary file 2 — Description of Additional Supplementary Files [file 41467_2023_43514_MOESM2_ESM.pdf]

Description of Additional Supplementary Files

File Name: NR-SAFE Study protocol
Description: Study protocol

File Name: Supplementary Data 1
Description: Study cohort

File Name: Supplementary Data 2
Description: Adverse events and vital signs

File Name: Supplementary Data 3
Description: Clinical laboratory values

File Name: Supplementary Data 4
Description: MDS-UPDRS analysis

File Name: Supplementary Data 5
Description: NADmed analysis

File Name: Supplementary Data 6
Description: LC-MS metabolomics

File Name: Supplementary Data 7
Description: Normality testing of data
