# Supplementary material for: NR-SAFE: a randomized, double-blind safety trial of high dose nicotinamide riboside in Parkinson’s disease
Source: Nat Commun. 2023 Nov 28;14:7793. doi: 10.1038/s41467-023-43514-6 (PMC10684646; doi:10.1038/s41467-023-43514-6)
Supplement: Supplementary file 10 — Reporting Summary [file 41467_2023_43514_MOESM10_ESM.pdf]

nature portfolio

Corresponding author(s): Prof. Charalampos Tzoulis

Last updated by author(s): 19.10.2023

Reporting Summary

Nature Portfolio wishes to improve the reproducibility of the work that we publish. This form provides structure for consistency and transparency in reporting. For further information on Nature Portfolio policies, see our Editorial Policies and the Editorial Policy Checklist.

Statistics

For all statistical analyses, confirm that the following items are present in the figure legend, table legend, main text, or Methods section.

n/a	Confirmed	
☐	☒	The exact sample size (*n*) for each experimental group/condition, given as a discrete number and unit of measurement
☐	☒	A statement on whether measurements were taken from distinct samples or whether the same sample was measured repeatedly
☐	☒	The statistical test(s) used AND whether they are one- or two-sided<br>*Only common tests should be described solely by name; describe more complex techniques in the Methods section.*
☐	☒	A description of all covariates tested
☐	☒	A description of any assumptions or corrections, such as tests of normality and adjustment for multiple comparisons
☐	☒	A full description of the statistical parameters including central tendency (e.g. means) or other basic estimates (e.g. regression coefficient) AND variation (e.g. standard deviation) or associated estimates of uncertainty (e.g. confidence intervals)
☐	☒	For null hypothesis testing, the test statistic (e.g. *F*, *t*, *r*) with confidence intervals, effect sizes, degrees of freedom and *P* value noted<br>*Give P values as exact values whenever suitable.*
☒	☐	For Bayesian analysis, information on the choice of priors and Markov chain Monte Carlo settings
☒	☐	For hierarchical and complex designs, identification of the appropriate level for tests and full reporting of outcomes
☐	☒	Estimates of effect sizes (e.g. Cohen's *d*, Pearson's *r*), indicating how they were calculated

Our web collection on statistics for biologists contains articles on many of the points above.

Software and code

Policy information about availability of computer code

Data collection	Data collection was performed using the electronic case report form www.viedoc.com, version 4.77.8648,13864.
Data analysis	Data analysis was performed using R version 4.2.2. (Vienna, Austria) using the following packages; tidyverse version 1.3.1, readxl version 1.4.0, openxlsx version 4.2.5, lubridate version 1.8.0, cowplot version 1.1.1, ggpubr version 0.4.0, ggsignif version 0.6.3, rvg version 0.3.2, officer version 0.6.0 and effsize version 0.8.1. The code required to reproduce the results presented in this manuscript have been deposited in the Neuromics Group repository https://git.app.uib.no/neuromics/nr-safe with no planned end date. Data can be accessed publicly by using the provided URL.

For manuscripts utilizing custom algorithms or software that are central to the research but not yet described in published literature, software must be made available to editors and reviewers. We strongly encourage code deposition in a community repository (e.g. GitHub). See the Nature Portfolio guidelines for submitting code & software for further information.

Data

Policy information about availability of data

All manuscripts must include a data availability statement. This statement should provide the following information, where applicable:

- Accession codes, unique identifiers, or web links for publicly available datasets
- A description of any restrictions on data availability
- For clinical datasets or third party data, please ensure that the statement adheres to our policy

Deidentified data of vital signs, MDS-UPDRS, NADMed analysis, and whole blood and urine LC-MS metabolomics required to reproduce the results presented in this manuscript have been deposited in the Neuromics Group repository https://git.app.uib.no/neuromics/nr-safe with no planned end date. Data can be accessed publicly by using the provided URL. The raw demographic, drug- and medical history data are protected and not available due to data privacy laws. Any additional information required to reanalyze the data reported in this paper is available from the corresponding authors upon request. Source data are provided with this paper. The study protocol has been provided as supplementary information.

Research involving human participants, their data, or biological material

Policy information about studies with human participants or human data. See also policy information about sex, gender (identity/presentation), and sexual orientation and race, ethnicity and racism.

Reporting on sex and gender	We reported on assigned sex from electronic medical records. We did not report on self-reported sex or gender. Apart from comparing sex in population characteristics to confirm even matching, no sex specific statistical analyses were performed.
Reporting on race, ethnicity, or other socially relevant groupings	This was not reported on.
Population characteristics	Population characteristics are described in detail in Table 2 of the manuscript.
Recruitment	Participants were recruited by giving information at patient organization meetings, by being asked for interest in participating in clinical trials in the outpatient clinic at the neurological department of Haukeland University Hospital, Bergen Norway, and by contacting patients who had previously requested to join a other clinical trials but had not met the inclusion or exclusion criteria for said trials. This could have caused the study to have self-selection bias where the participants generally have less disease burden both of Parkinsons disease and of other co-morbidities. This was however unlikely to have effected the results of the trial, as a large part of the participants had significant co-morbidities.
Ethics oversight	The trial follwed Good Clinical Practice (GCP) guidelines and had external monitoring. The trial was approved by The Regional Committee for Medical and Health Research Ethics, Western Norway (379218).

Note that full information on the approval of the study protocol must also be provided in the manuscript.

Field-specific reporting

Please select the one below that is the best fit for your research. If you are not sure, read the appropriate sections before making your selection.

☒ Life sciences ☐ Behavioural & social sciences ☐ Ecological, evolutionary & environmental sciences

For a reference copy of the document with all sections, see nature.com/documents/nr-reporting-summary-flat.pdf

Life sciences study design

All studies must disclose on these points even when the disclosure is negative.

Sample size	Sample size is n = 10 per group (n = 20 participants in total). The study did not include a power calculation as it was descriptive in character. A sample size of n = 10 per group was chosen as our previous study NADPARK (PMID: 35235774), testing 1000mg NR for 1 month, showed a homogenous metabolic response to NR. We therefore assumed this sample size would be sufficient to assess the short-term safety of an oral dose of 3000 mg NR.
Data exclusions	Single participants removed from analysis due to data entry error and not fasting for prequisite analyses. Specificed in text, tables and supplementary data.
Replication	Analyses in the study were not replicated.
Randomization	Randomization consisted of two blocks, each of block size 4. Allocation was 1:1. Randomization was performed by the electronic case report form www.viedoc.com.

Blinding	Both investigators and participants were masked to the intervention.

Reporting for specific materials, systems and methods

We require information from authors about some types of materials, experimental systems and methods used in many studies. Here, indicate whether each material, system or method listed is relevant to your study. If you are not sure if a list item applies to your research, read the appropriate section before selecting a response.

Materials & experimental systems

n/a	Involved in the study
☒	☐ Antibodies
☒	☐ Eukaryotic cell lines
☒	☐ Palaeontology and archaeology
☒	☐ Animals and other organisms
☐	☒ Clinical data
☒	☐ Dual use research of concern
☒	☐ Plants

Methods

n/a	Involved in the study
☒	☐ ChIP-seq
☒	☐ Flow cytometry
☒	☐ MRI-based neuroimaging

Clinical data

Policy information about clinical studies

All manuscripts should comply with the ICMJE guidelines for publication of clinical research and a completed CONSORT checklist must be included with all submissions.

Clinical trial registration	The trial was registered on ClinicalTrials.gov: NCT05344404.
Study protocol	The study protocol is supplied as supplementary information to the manuscript.
Data collection	Data was collected from participants in the period 29.04.22 01.07.22. Data was collected at the outpatient clinic at the Neurological department at Haukeland University Hospital, Bergen, Norway.
Outcomes	The primary outcome was the incidence of treatment-associated moderate and severe adverse events. This was assessed by general medical examination, measurement of vital signs, collection of safety routine blood biochemistry and screening for self- reported adverse events from participants. This was assessed at clinical visits on days 0, 7, 14, 21 and 28. Additional screening for self-reported adverse events was performed on days 3 and 35.<br><br>Secondary outcomes were between group differences in treatment associated mild adverse events, assessed on the same days as the primary outcome. Additional secondary outcomes were between-group differences in changes of the NAD metabolome in blood and urine, measured by liquid chromatography-mass spectrometry, and between-group differences in clinical severity of Parkinson's disease, measured by the Movement Disorders Society Unified Parkinson's Disease Rating Scale (MDS-UPDRS). Assessment and sample collection for this was perfomed at baseline and day 28.<br><br>Exploratory outcomes were between-group differences in change in homocysteine, fasting blood glucose and serum insulin levels.

Plants

Seed stocks	Not applicable.
Novel plant genotypes	Not applicable.
Authentication	Not applicable.
